# Interventional Endoscopic Ultrasonography: Advances in Application

**DOI:** 10.3390/jcm14103286

**Published:** 2025-05-08

**Authors:** Haidar Khan, Sharon Slomovich, Neal C. Shah, Frank Gress

**Affiliations:** Icahn School of Medicine at Mount Sinai South Nassau, Oceanside, NY 11572, USA; haidar.khan25@gmail.com (H.K.); sharon.slomovich@snch.org (S.S.); frank.gress@snch.org (F.G.)

**Keywords:** interventional EUS, pancreatic fluid collection [PFC], EUS-guided biliary drainage [EUS-BD], EUS-guided gallbladder drainage [EUS-GBD], EUS-guided gastroenterostomy [EUS-GE], lumen-apposing metal stent [LAMS], EUS-guided anastomosis

## Abstract

Technological advances have greatly expanded the diagnostic and therapeutic capabilities of endoscopic ultrasound (EUS). The integration of cutting-edge imaging techniques, including tissue harmonic echo, contrast-enhanced harmonic EUS, elastography, and needle-based confocal laser endomicroscopy, have significantly enhanced lesion characterization and diagnostic accuracy. Additionally, developments in stent design and the introduction of new accessories have broadened the therapeutic applications of EUS. Ongoing innovations in clinical practice have transformed procedures such as drainage, tumor ablation, EUS-directed transgastric endoscopic retrograde cholangiopancreatography (ERCP), the placement of fiducial markers, advancements in endohepatology, lesion characterization, and treatment. These developments continue to expand the role of EUS in delivering precise and effective therapeutic interventions.

## 1. Introduction

Interventional endoscopic ultrasonography [EUS] combines endosonographic with fluoroscopic-guided therapeutic endoscopy. Interventional EUS was developed over the last 20 years based on insights gained from endoscopic drainage of pancreatic fluid collections [PFCs] and further advanced by the advent of lumen-apposing metal stents [LAMS]. Drainage and anastomosis of the hepatobiliary and luminal tracts, targeted biopsies, endohepatology, and targeted tumor therapies are the procedures that comprise the field of interventional EUS. In this review, we focus on the latest advancements in interventional EUS, examining the most current data and their clinical implications.

## 2. EUS Anastomosis

Endoscopic ultrasound-guided gastrointestinal anastomosis was first described in swine models in 2002, when a modified 19G needle was used to introduce metal tags under EUS vision, resulting in suture apposition between lumens and posterior stent placement [1]. Endoscopic ultrasound-guided gastrojejunostomy [EUS-GJ] makes use of LAMS to circumvent both benign and malignant mechanical obstructions, including gastric outlet obstruction [GOO] afferent loop syndrome, as well as applying it to altered surgical anatomy, including Roux-en-Y gastric bypass.

There are many approaches, the most common being the direct or antegrade approach [2]. The direct method begins with the visualization of the jejunal loop under EUS guidance, which can be punctured and distended with a 19G or 22G needle for further confirmation. Once the appropriate site is determined, a hot LAMS is placed freehand to create the anastomosis. 

The wireless EUS-guided gastroenterostomy technique [WEST] is a device-assisted direct approach [3]. This procedure utilizes an oroduodenoal or orojejunal catheter to infuse contrast agents to improve EUS visibility of the jejunal loop. Additional device-assisted approaches utilize guidewires following the puncture of the jejunum to assist with LAMS placement.

Rarely, a retrograde approach can also be utilized when a linear echoendoscope is able to traverse the GOO. In this technique, the echoendoscope is placed in the distal duodenum, and the LAMS is deployed into the stomach. This technique is technically less demanding as the stomach is a larger target for LAMS deployment [4].

An alternative technique developed in Japan, called EUS-guided balloon-occluded gastrojejunostomy bypass [EPASS], utilizes a special device consisting of an enteric tube with two balloons [5]. In this technique, a guidewire is placed at maximal length into the small bowel under endoscopy. Then, the standard endoscope is exchanged over the guidewire and replaced with the double balloon tube until the balloons are in the duodenum and jejunum and inflated with saline to anchor the small bowel [5]. The remainder of this technique utilizes the antegrade approach, with the enteric tube serving as the conduit for contrast injection. The device utilized in EPASS is not commercially available in the United States, and the technique has largely fallen out of favor with the development of WEST [3].

A multicenter study demonstrated the high technical (92%) and clinical (88%) success of direct EUS-GJ anastomosis, with both freehand and device-assisted methods utilized [6]. EUS-GJ anastomosis was deemed appropriate for both benign and malignant GOO. The efficacy is comparable to surgical intervention; however, no head-to-head studies exist.

In a global study of 467 patients, EUS-gastroenterostomy [EUS-GE] stent misdeployment was shown to occur in 9.85% of cases. Misdeployment was classified to be fatal [2.2%], severe [13%], moderate [23.9%], or mild [60.9%]. The four types of stent misdeployment identified were Type I [peritoneal distal flange, proximal flange in the stomach without enterotomy, 63.1%], Type II [with enterotomy, 30.4%], Type III [small bowel distal flange, 2.2%], and Type IV [colon, 4.3%]. Eleven percent of cases required surgery, with the remainder being treated endoscopically [7]. This highlights the expertise required to appropriately identify the jejunal loop on EUS, as well as the technical skill necessary to deploy the LAMS.

All patients receiving EUS-GE, independent of technique, are recommended to undergo inpatient observation and receive a 7-day course of broad spectrum antibiotics. Though there is no consensus on timing, the LAMS should be exchanged in patients with a greater-than-six-month life expectancy [3] (Table 1).

## 3. Pancreatic Drainage

EUS-guided drainage has emerged as the recommended technique for pancreatic pseudocysts and walled off pancreatic necrosis [WOPN], providing a minimally invasive yet highly effective option [8,9]. To achieve the greatest results, addressing walled off pancreatic collections requires a multidisciplinary approach. A retrospective analysis reveals that step-up therapy may be beneficial in cases of larger collections, paracolic extension, or substantial necrosis. To assess crucial characteristics, such as size, position, wall maturity, intracavitary debris, proximity to the gastrointestinal wall, and the presence of vascular structures, preprocedural imaging with contrast-enhanced CT or MRI is recommended. Various drainage methods, such as plastic pigtail stents, self-expanding metal stents [SEMS], and lumen-apposing metal stents [LAMS], are detailed, with LAMS being the most extensively utilized and considered the standard by many advanced endoscopists [10]. Numerous studies have demonstrated the effectiveness of LAMS in controlling PFCs and WOPN [11,12,13,14,15,16,17].

LAMS placement for WOPN can be performed using two techniques. Non-cautery-enhanced LAMS involves several steps: EUS is used to identify a suitable drainage site near the gastric or duodenal wall, followed by needle puncture with a 19 gauge fine-needle aspiration [FNA] needle, fluid aspiration [if needed], guidewire placement, and tract dilation [18]. Larger dilation is required for plastic stents, while smaller dilation works for metal stents or cold LAMS [18]. A stent is then placed, and endoscopic necrosectomy can be performed during the same or a separate session. Cautery-enhanced LAMS, on the other hand, simplifies the process with a single-step approach. The device uses a cauterized needle to directly access the WOPN, bypassing the need for guidewires, tract dilation, or fluoroscopy. Both techniques are effective, with the choice depending on patient needs and available tools [18].

Pseudoaneurysm formation is a potentially fatal complication which may form from the pancreatitis but can also occur secondarily to vascular trauma during endoscopic necrosectomy or percutaneous drain placement [19]. Given the high risk for spontaneous rupture, patients typically require embolization therapy prior to further drainage [19].

A multiple transluminal gateway approach is another option for draining complex multiloculated pancreatic collections. This procedure involves placing several plastic pigtail stents or LAMS at various access locations transmurally across the stomach or duodenum into the pancreatic collection, providing for many drainage sites. While only a few facilities use this approach, it could enhance necrotic material drainage and eliminate the need for high-risk surgeries such as necrosectomy [8,20]. Some endoscopists will use this technique on larger collections [>12 cm]; however, larger and non-necrotic collections may drain better with single access, and the drainage of complex minor lesions can also benefit from this approach [21]. Additional investigation is required for the proper use of this approach.

## 4. Gallbladder Drainage

Endoscopic ultrasound-guided gallbladder drainage [EUS-GBD] is a minimally invasive approach for treating a range of gallbladder illnesses, particularly when more risky or impracticable traditional procedures, such as surgery or percutaneous gallbladder drainage [PT-GBD], are required [22]. This strategy combines the benefits of EUS imaging for gallbladder vision with the ability to rapidly access and empty the gallbladder via the duodenum or stomach by coiling a guidewire into it for stent deployment.

Endoscopic, endosonographic, and fluoroscopic guidance is used to access the gallbladder, which is usually conducted through the prepyloric antrum or duodenum [22]. Color Doppler imaging is used to avoid interfering arteries and minimize bleeding hazards [22]. The first method involves puncturing the gastrointestinal lumen with a FNA needle, confirming gallbladder access with bile aspiration, and inserting a stent utilizing guidewires and dilation methods [22]. Alternatively, a single-step technique using a cautery-enhanced delivery device, such as the AXIOS stent and the electrocautery-enhanced delivery system [Axios-EC], punctures the gallbladder and installs the stent under EUS guidance, which is appropriate for gallbladders that are close together [22]. To prevent stent occlusion and problems, a plastic double-pigtail stent is frequently put inside the metal stent [22]. 

EUS-GBD offers several advantages over traditional surgical gallbladder removal for acute cholecystitis. EUS-GBD improves the drainage of inflamed gallbladder contents, which helps to relieve symptoms and prevent potential complications from gallbladder disease. Patients who are not ideal candidates for typical surgical procedures can benefit from this minimally invasive method, which allows for shorter hospital stays, better results, and the avoidance of larger incisions and general anesthesia [22,23]. Furthermore, EUS-guided gallbladder drainage has advantages over the traditional non-surgical treatment method, PT-GBD, which is associated with a number of adverse events, including bowel perforation, pneumothorax, bleeding, bile leaks, secondary infections, discomfort, and the inability to be performed on patients on anticoagulation or with severe ascites [22,24,25].

Some individuals choose to have a cholecystectomy after EUS-GBD. This allows for a more efficient surgery time. Future consensus recommendations among advanced endoscopists and surgeons are required due to the growing number of bridging therapy cases [25,26,27].

Although EUS-GBD is a relatively new treatment, investigations have yielded promising findings in terms of efficacy and safety [28,29]. Despite the procedure’s perceived relative safety, reported complications include bile leakage, stent migration into the gallbladder or peritoneum, gastroduodenal perforation, hemorrhage, pneumoperitoneum, and even recurring acute cholecystitis caused by stent occlusion [23] (Table 2).

## 5. Biliary Drainage

ERCP-guided transpapillary access and the placement of SEMS are currently the standard of care for biliary decompression [30,31]. However, there is a risk of early adverse events such as acute pancreatitis and acute cholecystitis following ERCP with SEMS placement [32,33]. In cases of malignant biliary obstruction, anatomical distortion can make ERCP difficult or impossible.

Choledochoduodenostomy [CDS] is a common EUS-guided biliary decompression procedure that entails creating a transmural biliodigestive fistula between the duodenal lumen and the proximal bile duct by inserting a stent with a SEMS or a LAMS, as a recent meta-analysis showed that rates of clinical and technical success, post-procedure adverse events, and reintervention were similar between the two types of stents [34]. Hepaticogastrostomy [EUS-HGS] involves the formation of a transmural fistula between the gastric lumen and the intrahepatic bile duct [35]. Because the ampulla is bypassed, EUS-guided biliary access has a very low to nearly nonexistent risk of procedure-related pancreatitis compared to ERCP [35].

Endoscopists are seeing more and more post-bariatric surgery anatomy as obesity rates in the United States climb. Patients with Roux-en-Y anatomy can more easily access the excluded stomach, duodenum, and biliary tree using the EUS-directed transgastric endoscopic retrograde cholangiopancreatography [EDGE] procedure. An EUS-guided LAMS is first inserted from the gastric pouch or proximal jejunum into the excluded stomach as part of the EDGE procedure. Next, the LAMS is traversed using an ERCP scope to perform therapeutic or diagnostic procedures. Lastly, the LAMS is removed, and the fistula is closed.

EDGE offers shorter procedure times and a higher technical success rate while maintaining a similar safety profile when compared to enteroscopy and laparoscopy assisted ERCP [36]. A recent meta-analysis showed EDGE as a more cost-effective option than laparoscopy-assisted [LA]-ERCP. While enteroscopy-assisted [EA]-ERCP had the lowest adverse event rate [8.4%, *p*  =  0.001], EDGE and LA-ERCP had similar rates [21.9% vs. 17.4%, *p*  =  0.32] [37]. EDGE achieved comparable technical [96.5% vs. 95%, *p*  =  0.98] and clinical success rates [96% vs. 93%, *p*  =  0.65] to LA-ERCP and outperformed EA-ERCP in terms of both technical [96% vs. 71%, *p*  =  0.01] and clinical success [96% vs. 59%, *p*  =  0.001] [37].

Stent dislodgement is the most frequent problem, and persistence of the fistula is another frequent consequence, which often resolves on its own or requires additional endoscopic procedures [36]. A single gastro-gastric fistula session, comprising the use of the LAMS suturing technique and either delayed ERCP or ERCP, can be used to treat EDGE patients to reduce dislodgement. A persistent fistula can occur in up to 10% of individuals, which can result in issues like ulceration, gastrointestinal discomfort, or weight gain. Most fistulas are treatable with endoscopic closure; however, bigger diameters and longer indwelling LAMS times are risk concerns.

The indication determines when ERCP should be performed; in emergency situations, the fistula can be traversed right away following LAMS implantation. Depending on the urgency, it is advised to give the fistula 5–14 days to mature for nonurgent causes to prevent dislodgement [36].

It has been estimated that the fistula will fully mature in around four weeks. There is the least chance of perforation and dislodgement when the LAMS is removed at this time. The closure method depends on the endoscopist’s choice; if fistula maintenance is required, LAMS are frequently swapped out for plastic double-pigtail catheters.

## 6. Intraabdominal Abscess Drainage

The methods typically used for draining pancreatic fluid collections and blocked off necrosis after pancreatitis are applied in EUS-guided abscess drainage [EUS-AD]. Under EUS supervision, a plastic stent, nasobiliary tube, or needle is introduced into the abscess for drainage once an endoscope has been advanced to the target location [38].

Currently, the standard treatment for intra-abdominal abscesses is percutaneous drainage with CT guidance. Surgery may be necessary for large abscesses near venous structures, and percutaneous drains may cause discomfort for patients. EUS-AD provides an alternative.

A recent meta-analysis evaluated the clinical and technical success rates of EUS-AD. They found a pooled clinical success rate of 90% [95% confidence interval [CI], 0.85–0.95], a technical success rate of 99% [95% CI, 0.97–1.00], and a recurrence rate of 1% [95% CI, 0.00–0.03]. Three of the eight studies reported complications of bleeding, perforation, and stent migration [39].

The procedure for EUS-guided liver abscess drainage [40,41,42,43,44,45,46,47,48,49,50,51,52,53,54,55,56] is the same as that for peripancreatic fluid collection and is commonly attempted after failed percutaneous drainage. EUS-guided drainage reduces the risk of infection, allows for clear doppler artery visualization, and provides direct trans-gastric access to left lobe liver abscesses. Although there are limited reports for drainage from the right lobe, it does appear feasible. For some patients, a brief trans-gastric route is considered safe.

Pelvic abscesses occur after surgery or in illnesses such as Crohn’s disease, and diverticulitis and are difficult to treat due to their anatomical location. There are limited documented studies in the current literature [57,58,59,60,61,62,63,64,65,66,67,68,69,70,71]. Current treatment options include surgery, percutaneous drainage, and EUS-guided drainage. Several EUS-guided procedures are documented, including needle aspiration, tract dilation, and stent implantation; nevertheless, each method frequently employs double-pigtail stents [57,58,59]. Short stents are desirable, particularly for lower rectum drainage, to reduce the discomfort caused by contact with the anal canal [69].

If not treated promptly, mediastinal abscesses can be fatal. While surgery is the preferred treatment, EUS-guided fine-needle aspiration using a transesophageal technique has proven diagnostically useful [72,73,74]. However, reports on EUS-guided drainage remain limited [75,76,77,78,79,80,81]. EUS-guided external drainage of mediastinal abscesses has numerous advantages. It facilitates irrigation and drainage, prevents failures by tube repositioning, and allows for recurrent culture samples. External drainage causes less pharyngeal discomfort than internal techniques and is easier to perform in narrow locations near the upper esophageal sphincter. Furthermore, patients can tolerate an oral elemental diet monitored by the nasobiliary drainage tube, although the fundamental limitation is the diameter, which can result in drainage failure. This limitation does not exist with LAMS placement.

## 7. Liver Biopsy

Liver biopsy remains the gold standard for assessing fibrosis and inflammation. EUS-guided liver biopsy (EUS-LB) offers distinct advantages over percutaneous and transjugular methods, including the ability to obtain tissue samples from both the left and right liver lobes in a single session while providing high-resolution imaging during the procedure.

This technique involves EUS visualization of the left lobe from the proximal stomach or the right lobe from the duodenal bulb. A color Doppler is then used to identify the safest needle trajectory, followed by the collection of tissue samples using a 19-gauge core biopsy needle. The most common complications include mild bleeding and subcapsular hematoma formation; however, the overall rate of adverse events is comparable to that of alternative approaches [82].

In terms of tissue acquisition, EUS-LB appears to yield results similar to other biopsy techniques [82]. As this procedure continues to evolve, it is expected that increasing familiarity among endoscopists will further enhance diagnostic accuracy and reduce complication rates.

## 8. Portal Pressure Gradient

Portal pressure gradient (PPG) measurement is considered the most reliable prognostic indicator in liver disease [83]. Traditionally, it is assessed using a transjugular approach, where the hepatic vein pressure is measured, and the free hepatic vein pressure is subtracted from the wedged hepatic venous pressure to estimate the PPG. While this method provides an accurate surrogate measurement for patients with sinusoidal portal hypertension, direct portal venous pressure measurement is necessary for improved accuracy in cases with different underlying causes of portal hypertension.

A recent prospective study investigated the correlation between the endoscopic ultrasound-guided portal pressure gradient (EUS-PPG) and the hepatic venous pressure gradient (HVPG) in patients with chronic portal hypertension. This single-center trial demonstrated comparable technical success rates for both EUS-PPG and HVPG (93.9% for each). The study, involving 33 patients, found a strong correlation between the two measurement techniques, as indicated by an intraclass correlation coefficient (ICC) of 0.82 (95% CI, 0.65–0.91). While this suggests that EUS-PPG could serve as a valid alternative to HVPG for assessing portal pressure, the authors did note that four patients (13.3%) exhibited discrepancies of ≥5 mmHg between the two methods. This study concluded that EUS-PPG represents a safe and reliable approach for direct portal pressure measurement in this patient population [84].

EUS-guided PPG (EUS-PPG) enables direct measurement of portal venous pressure, eliminating the need for indirect calculations or estimations. This technique involves EUS visualization of the hepatic and portal veins, followed by pressure measurement using either a 25-gauge needle with a manometer/pressure transducer or a 22-gauge needle connected to a central venous pressure monitor. The PPG is then determined by subtracting the hepatic venous pressure from the portal venous pressure [83]. Some studies recommend averaging three separate measurements to enhance accuracy [85]. While no adverse events have been reported in current studies, the sample sizes are limited, and procedures have been performed by highly experienced endoscopists [85].

EUS-PPG has the potential to become the new gold standard for evaluating liver disease by enabling PPG measurements across various etiologies, overcoming the limitations of traditional methods that are primarily effective for sinusoidal disease.

## 9. Variceal Embolization

The management of gastric varices (GVs) includes medical treatment, endoscopic interventions, and interventional radiology (IR)-guided procedures such as transjugular intrahepatic portosystemic shunt (TIPS) and balloon retrograde transvenous obliteration (BRTO). Traditional endoscopic approaches involve the injection of thrombosis-inducing agents, such as acrylate glue or thrombin, directly into the varices to promote clot formation.

This procedure typically involves EUS visualization of the GVs combined with Doppler imaging to guide the targeted injection of acrylate glue or thrombin, followed by a repeat Doppler examination to confirm reduced blood flow. A newer approach incorporates coil injection using a 19-gauge or 22-gauge needle in a similar manner. However, this technique introduces additional risks, including thrombus formation, embolization, and a higher likelihood of rebleeding compared to TIPS or BRTO [86].

Currently, the primary role of EUS-guided therapy is as a salvage option for patients who are not candidates for IR-guided procedures or as a bridging therapy for stable patients. However, due to the limited availability of data and formal guidelines on GV management, the role of EUS in this setting may expand in the future (Table 3).

## 10. Liver Elastography

Transient elastography with FibroScan™ is a widely utilized non-invasive method for evaluation of liver fibrosis [87]. However, factors such as ascites and large body habitus—both common in patients with liver disease—can hinder the accuracy of transabdominal imaging.

EUS-guided real-time elastography (EUS-RTE) is a recently introduced technique for evaluating hepatocyte compressibility. The procedural approach closely resembles the standard EUS of the liver. EUS-RTE determines tissue elasticity using either strain-based or shear wave methods. The strain method assesses differences in tissue distortion following gentle probe pressure, along with physiological pulsations and respiratory-induced movement, to calculate elastography [88]. In contrast, the shear wave method measures the velocity of acoustic radiation force-generated waves to quantify liver stiffness [88].

The risks associated with EUS-RTE are comparable to those of conventional EUS. Current evidence is promising, demonstrating that EUS-RTE accurately assesses liver fibrosis indices in patients with metabolic liver disease, cirrhosis, and even those without liver disease [88].

## 11. Tissue Harmonic Echo

Endoscopic ultrasound tissue harmonic echo (EUS-THE) is an imaging technique that enhances visualization of the gastrointestinal tract by utilizing harmonic frequencies generated as ultrasound waves pass through tissue. Compared to conventional ultrasound, EUS-THE offers improved image resolution and reduced noise, making it a valuable tool for lesion characterization [89].

Originally applied in transabdominal ultrasonography, EUS-THE operates on the principle that ultrasound waves produce harmonic frequencies at twice their original frequency as they travel through tissue, leading to clearer and more detailed imaging [90]. This technique is particularly beneficial for assessing pancreatic and biliary lesions that are challenging to detect with standard ultrasound methods [91,92]. The introduction of advanced endoscopic ultrasound processors, such as the EU-ME2 Premier Plus (Olympus Medical Systems Corp., Tokyo, Japan), has further refined the imaging quality of both solid and cystic pancreatic lesions [92].

Over the past two decades, numerous studies have explored the role of EUS-THE in diagnosing pancreatic diseases [89,91]. A retrospective study analyzing 50 patients with pancreatic lesions (38 cystic, 12 solid) compared EUS-THE with conventional B-mode imaging. The findings demonstrated that THE mode provided superior lesion characterization, particularly for pancreatic cystic lesions; however, this study is limited given its retrospective design, it being performed at a single center with a small number of observers, and the fact that only highly experienced endosonographers were enlisted [91].

While EUS-THE has shown great promise in enhancing lesion visualization, further research is necessary to determine its efficacy in distinguishing between benign and malignant changes, as well as its potential role in tumor staging [93].

## 12. Contrast-Enhanced Harmonic EUS

Contrast-enhanced harmonic endoscopic ultrasound (CEH-EUS) is an advanced imaging technique that integrates tissue harmonic imaging with contrast agents to enhance the visualization of vascular structures and tissue perfusion [94,95].

This method involves the administration of microbubbles, gas-filled particles that circulate in the bloodstream, followed by harmonic imaging during EUS [96]. These microbubbles act as contrast agents, improving the delineation of blood vessels and aiding in the characterization of focal lesions in organs such as the pancreas and lymph nodes [97,98,99]. Unlike Doppler imaging, which primarily detects larger vessels, CEH-EUS provides detailed visualization of the microvasculature, offering a more comprehensive assessment of lesion vascularity [100]. Its function is similar to contrast-enhanced CT (CE-CT); however, CEH-EUS can complement CT by capturing high-resolution images of small nodular lesions that may be difficult to detect with CT alone.

The use of contrast agents in CEH-EUS has been shown to enhance the diagnostic accuracy of EUS in differentiating benign from malignant lesions and in staging gastrointestinal and pancreaticobiliary tumors [94,100]. Additionally, CEH-EUS has been proposed to improve the efficacy of fine-needle aspiration (FNA) over conventional EUS by providing clearer visualization of small and subtle lesions. By enhancing the visibility of vascular structures and lesion borders, CEH-EUS allows endoscopists to better target viable tissue while avoiding necrotic areas and blood vessels during FNA [101].

However, the benefits of CEH-EUS over standard EUS-FNA remain a topic of debate. Several studies, including two randomized controlled trials, have yielded conflicting results regarding its advantage in tissue acquisition [101,102,103,104,105]. A recent study by Facciorusso et al. found no significant difference in pancreatic mass tissue acquisition between CEH-EUS-guided FNA and conventional EUS-FNA [106]. Therefore, larger randomized controlled trials are needed to further evaluate the clinical impact of CEH-EUS-guided FNA compared to standard EUS-FNA in pancreatic tissue sampling.

## 13. Pancreatic Elastography

By analyzing changes in tissue elasticity, endoscopic ultrasound elastography (EUS-E) can differentiate between normal and abnormal tissues, identify areas of fibrosis or tumor infiltration, and guide treatment decisions. EUS-E has been primarily studied in the assessment of pancreatic pathologies and lymph nodes [107]. This technique has improved the diagnosis of chronic pancreatitis, aided in distinguishing solid pancreatic lesions (benign versus malignant), and helped to evaluate the malignant potential of lymph nodes [108,109,110,111].

EUS-E is also expanding into applications for the liver, biliary system, and gastrointestinal tract [112,113,114]. As a minimally invasive method for assessing tissue stiffness, EUS-E enhances conventional EUS imaging, providing valuable diagnostic insights. With ongoing research and technological advancements, its role is expected to continue growing, improving diagnostic accuracy and patient management across various gastrointestinal and hepatobiliary conditions.

## 14. Needle-Based Confocal Laser Endomicroscopy

Needle-based confocal laser endomicroscopy (nCLE) is an advanced endoscopic imaging technique that enables high-magnification, high-resolution imaging in real time. This technology provides optical histology at the microscopic level, potentially overcoming the limitations of fine-needle aspiration (FNA), such as sampling errors, the lack of on-site cytopathologists in many centers, and non-diagnostic specimens [115,116].

nCLE is performed by passing a probe through a 19-gauge FNA needle to visualize the pancreatic cyst epithelium at a cellular level. Several clinical trials have investigated its diagnostic potential. The INSPECT trial evaluated nCLE in combination with other imaging modalities, such as magnetic resonance imaging (MRI) and computed tomography (CT), to assess its ability to aid in the early identification of pancreatic cysts [117]. Eight referral centers, with a total of 66 patients, performed nCLE with preliminary data, suggesting that nCLE has high specificity in the detection of PCN but may be limited by low sensitivity. However, the safety of nCLE requires further evaluation [117]. The DETECT trial was a prospective feasibility study examining 37 patients undergoing nCLE as a stand-alone test and in combination with cystoscopy using a through-the-needle fiberoptic probe under EUS guidance, demonstrating that the procedure is technically feasible, safe, and sensitive [118].

Studies have shown that while nCLE criteria have a high specificity (>80%), sensitivity remains lower and somewhat variable [119,120,121,122]. The CONCYST-01 trial, which enrolled 67 patients, with 56 followed for at least 12 months, found that nCLE correlated with the final diagnosis in 77% of cases, surpassing the accuracy of cytology or carcinoembryonic antigen (CEA) testing (71%) [123]. Furthermore, nCLE has shown the ability to precisely differentiate between various subtypes of pancreatic cystic neoplasms (PCNs) [124]. As research continues, nCLE may become an integral tool for improving the diagnosis and management of pancreatic cystic lesions.

## 15. Fiducial Needles

Fiducial needles function as markers to enable precise radiation therapy or surgical procedures while minimizing harm to surrounding structures. Historically, their placement was limited to percutaneous or surgical methods. Pancreatic cancer treatment, in particular, benefits from fiducial markers, as stereotactic body radiation therapy requires high doses that pose risks to adjacent organs [125]. However, many pancreatic lesions are difficult to access through percutaneous or surgical approaches.

EUS-guided fiducial needle placement utilizes a fine-needle aspiration (FNA) needle, typically 19-gauge or 22-gauge, depending on the size of the fiducial marker. Two main techniques are used, namely, front loading and back loading.

In the front-loading method, the EUS-FNA needle is inserted into the target lesion, the stylet is removed, and the fiducial marker is advanced through the needle’s lumen before being positioned with the stylet. However, this technique has drawbacks, including difficulty in maneuvering angulated FNA needles and the potential introduction of air during stylet reinsertion, which may obscure imaging [125].

The back-loading approach involves retracting the stylet by approximately 1 cm, placing the fiducial marker into the FNA needle, and sealing it with bone wax [126]. This method helps prevent air introduction and reduces procedure time. However, it also presents challenges, such as the risk of needlestick injuries and fiducial marker migration. 

With EUS-guided placement gaining traction as a reliable technique, preloaded fiducial needle systems are being developed to enhance efficiency and safety (Table 4).

## 16. Tumor Ablation

EUS-guided tumor ablation (EUS-TA) provides a minimally invasive alternative to surgery by enabling the direct visualization and targeted treatment of localized tumors within the gastrointestinal tract. This approach is generally classified into direct and indirect therapies.

Direct therapies encompass techniques such as radiofrequency ablation (RFA), ethanol injections, photodynamic therapy, laser ablation, cryotherapy, and brachytherapy. Indirect methods involve the fine-needle injection of antitumor agents or the placement of fiducial markers, as previously discussed.

A recent meta-analysis reviewing 13 studies assessed the efficacy of EUS-RFA in patients with unresectable pancreatic ductal adenocarcinoma. The findings demonstrated a high technical success rate (100%) and clinical success rate (91.5%), with abdominal pain (9.82%) being the most frequently reported adverse event. These results suggest that EUS-RFA is a promising therapeutic option; however, it is important to acknowledge that most studies included in this analysis were single-center studies with small sample sizes [127].

Furthermore, recent research has explored the application of EUS-RFA in treating neuroendocrine tumors and pancreatic cystic neoplasms. A multi-center pilot safety feasibility study, although low-powered, with only eight patients, showed the technique to be safe and well tolerated, with response rates varying from complete tumor resolution to a 50% reduction in tumor size [128].

## 17. Conclusions

Interventional EUS allows for a minimally invasive solution to several clinical scenarios. In the past two decades, it has emerged as the preferred method of diagnosing and managing various pancreaticobiliary diseases. With new advances in the field, interventional EUS demonstrates a promising role in other avenues such as anastomoses, hepatology, and cancer treatment.

## Figures and Tables

**Table 1:** Summary of EUS-GJ anastomotic approaches.

Technique	Benefits	Drawbacks
Freehand	Largest pool of data on efficacy and outcomes; faster procedure time	High operator skill required; increased difficulty in assessing jejunal loop
WEST	Improved visualization of the jejunal loop; reduced risk of misdeployment	Longer procedure time; catheter must traverse obstruction
EPASS	Improved visualization and stabilization of jejunal loop	Not practiced outside of Asia; longer procedure time than WEST
Guidewire	Improved visualization of the jejunal loop; reduced risk of misdeployment	Guidewire must traverse obstruction
Retrograde	Fastest procedure; lowest risk for misdeployment	Echoendoscope must traverse obstruction

**Table 2:** Summary of EUS-GBD approaches.

Technique	Summary
Cautery-enhanced LAMS	Single step procedure; lower adverse event rate
“Cold” LAMS	Longer procedure time; risk of guidewire loss; requires tract dilation
SEMS	Longer stent patency; higher risk of migration; poor apposition prevents fistula formation

**Table 3:** Summary of gastric varices management options.

Technique	Benefits	Drawbacks
Transjugular Intrahepatic Portosystemic Shunt (TIPS)	Reduced portal hypertension; can be used in patients with portal vein thrombus; treats ascites; readily available at most centers	Increased hepatic encephalopathy; worsens or induces heart failure and liver failure
Balloon Retrograde Transvenous Obliteration (BRTO)	Effective in preventing rebleeding; safely performed in patients with poor hepatic reserve; improves hepatic encephalopathy	Requires gastrorenal shunt; increases portal hypertension; worsens ascites; contraindicated in patients with portal vein thrombus; limited to expert centers
Endoscopic Ultrasound Variceal Embolization	Improved visualization; doppler flow assessment; reduced rebleeding rates; combined approach with cyanoacrylate glue and coil deployment	Rebleeding rate higher compared to IR techniques; embolization risk (less in combined glue and coil technique and with reduced glue volume); limited to expert centers

**Table 4:** Summary of approaches for EUS-guided fiducial needle placement.

Approach	Benefits	Drawbacks
Front Loading	Direct placement of marker through needle lumen; familiar technique	Difficult maneuverability with angulated FNA needles; air introduction from stylet reinsertion may obscure imaging
Back Loading	Reduces air introduction; shorter procedure time	Risk of needlestick injuries; possibility of fiducial marker migration
